# Setting Up a Teleneurology Clinic during COVID-19 Pandemic: Experience from an Academic Practice

**DOI:** 10.1155/2022/4776328

**Published:** 2022-01-18

**Authors:** Nakul Katyal, Naureen Narula, Raghav Govindarajan, Pradeep Sahota

**Affiliations:** ^1^Department of Neurology, University of Missouri, Columbia, USA; ^2^Department of Pulmonology and Critical Care Medicine, Staten Island University Hospital, New York, USA

## Abstract

The declaration of the COVID-19 pandemic necessitated rapid implementation of telehealth across all neurological subspecialties. Transitioning to telehealth technology can be challenging for physicians and health care facilities with no prior experience. Here, we describe our experience at the Neurology and Sleep Disorders Clinic at the University of Missouri-Columbia of successful transition of all in-person clinic visits to telehealth visits within a span of 2 weeks with a collaborative effort of clinic staff and the leadership. Within a month of launch, 18 clinic providers with no prior telehealth experience conducted 1451 telehealth visits, which was the 2nd highest number of telehealth visits conducted by any department at the University of Missouri-Columbia Health Care system. Lack of connectivity, poor video/audio quality, and unavailability of smart devices among rural populations were the important shortcomings identified during our telehealth experience. Our study highlighted the need for expansion of high-speed internet access across rural Missouri. We hope our experience will help other health care facilities to learn and incorporate telehealth technology at their facilities, overcome the associated challenges, and serve patient needs while limiting the spread of the COVID-19.

## 1. Introduction

The role of telemedicine in neurology has been well documented in providing prompt assessment and treatment of acute stroke [1]. The application of teleneurology across other neurologic subspecialties, however, remains sparsely reported [2–4]. Limitations with neurological examination, technical and connectivity issues have been identified as key factors limiting widespread use of teleneurology [3–10]. With dramatic changes in circumstances following the declaration of the COVID-19 pandemic and to limit the spread of SARS-CoV-2 infection, rapid implementation of teleneurology across all neurological subspecialties became paramount [3, 11–13]. Here, we describe our experience of successful transition of in-person clinic visits to telehealth visits (and eventually to a hybrid model) following declaration of COVID-19 pandemic. The Neurology and Sleep Disorders Clinic at the University of Missouri-Columbia conducted over 1400 telehealth visits within one month with no prior experience of doing telehealth clinics. We discuss the key components to our successful transition, associated challenges with potential solutions, and future role of telehealth visits in advancing care of patients with neurological diseases. Our experience, we hope, may be of value to other health care facilities to learn, implement, incorporate, and further explore telehealth technology at their facilities to cater to patient care needs safely while limiting the spread of the COVID-19.

The state of Missouri reported its first case of COVID-19 on March 6, 2020 [14]. The number of cases continued to rise steadily over the next few weeks. Boone county reported the first case of COVID-19 on March 17 [15]. On the same day, the chair of the Department of Neurology at the University of Missouri-Columbia decided to discontinue all in-person neurology clinic visits in order to protect the health of patients and health care workers. To limit the spread of the virus, the leadership decided to equip the Neurology and Sleep Disorders Clinic with capability to convert all in-person clinic visits to telehealth visits by the first week of April, 2020. Such telehealth visits were never conducted before at our clinic. The declaration was followed by a challenging task of training the clinic staff and physicians for their first ever telehealth experience.

**Figure 1:**
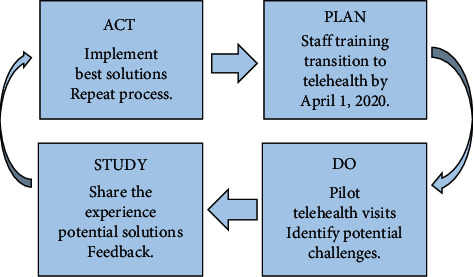
Plan Do Study Act model to train clinical faculty physicians and trainees.

The Neurology and Sleep Disorder Clinic at the University of Missouri-Columbia comprises 10 subspecialties served by 18 faculty physicians, 21 residents, and 7 fellows. Taking the lead, the chair of the department along with the associate medical director familiarize themselves with Zoom secure meetings, an existing secure telehealth platform used by the University of Missouri Health Care. After completing the training, they implemented the Plan Do Study Act (PDSA) model to train other clinical faculty physicians and trainees (Figure 1). Leading with an example, the chair of the department conducted several telehealth clinic visits for sleep disorder patients, a week before the launch date, and identified potential challenges and shared his experience with other providers.

### 1.1. Process

To enable rapid and successful launch of telehealth video visits, we focused on 4 key components: (1) training the patient service representatives (PSRs), (2) training the clinic nursing staff, (3) training the physicians and trainees including residents and fellows, and (4) ensuring adequate software and hardware support.

## 2. Training the Patient Service Representatives (PSRs)

**Figure 2:**
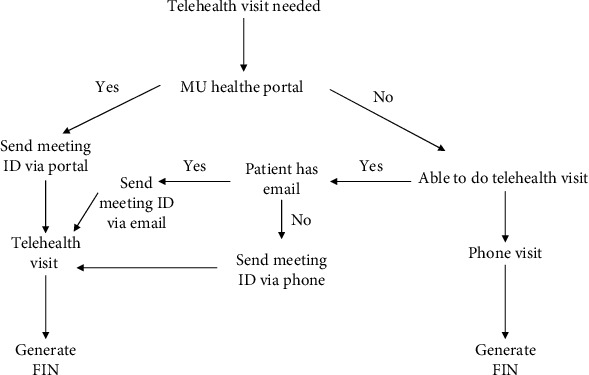
The workflow process for scheduling a telehealth visit by patient safety representatives (PSRs).

PSRs were responsible for triaging, scheduling, or rescheduling patient visits. The PSRs underwent online training provided by the University of Missouri Health Care system to familiarize themselves with patient eligibility criteria for telehealth visits, triaging, and scheduling processes. After completing the online training, the PSRs formed 2 groups and implemented teach back techniques to consolidate the learned objectives. The PSR teams were provided with a checklist of questions and process instructions for conducting a phone call with patients. The PSRs were responsible for calling all patients identified as appropriate for telehealth visit to assess their ability to carry out telehealth visit appointments (availability of smartphone/tablet/computer with camera) and then educate patients on instructions for successful telehealth appointment. The University of Missouri uses the “MU Health e” (patient portal) application, which interfaces with the patient's electronic medical records for clinic notes, labs, imaging, and communication with the care team. If the patient agreed to a video visit, they sent the meeting ID (unique 9-digit code) and instructions via the secure portal regarding the process and regulations. If the patient did not have a MU Health e portal, they were offered one and then provided the instructions via safe portal. If the patient refused the telehealth appointment for any reason (did not have a smartphone and did not feel equipped to do a telehealth visit), they were offered a phone visit. After confirming the type of visit, the PSR would check in the patient and generate a Facility Identification Number (FIN) for the telehealth appointment. Each return appointment was scheduled for 30 minutes and new appointments for 1 hour. The workflow process for scheduling a telehealth visit by PSR is depicted in Figure 2.

## 3. Training the Nursing Staff

**Figure 3:**
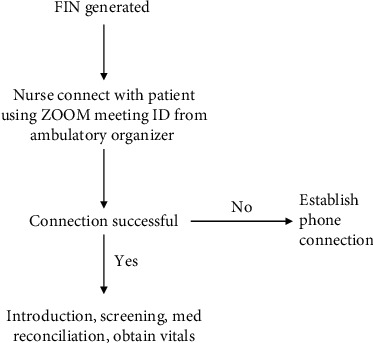
The workflow process for previsit nursing screening process.

The nursing staff underwent online training provided by the University of Missouri Health Care system to familiarize themselves with the HIPAA and security protocol with telehealth visits and previsit screening processes. After completing the online training, the nursing staff also formed 2 groups and implemented teach back techniques to consolidate the learned objectives. The nursing teams were provided with a checklist of questions and process instructions for conducting a phone call with patients. Nursing staff was responsible for calling patients via Zoom meeting ID or via phone call as directed by the PSR staff. The staff would connect with patients, a day prior to their clinic visit. They would conduct screening, complete medication reconciliation, and educate patients on best practices for sound and lighting optimization during telehealth appointments. The workflow process for previsit nursing screening process is depicted in Figure 3.

## 4. Training the Physicians

The change in circumstances surrounding COVID-19 pandemic instilled a sense of urgency among physicians to learn and get accustomed to a new platform of physician-patient virtual interaction [3]. After completing the online training, the chair of department and the associate medical director implemented a teach back technique and used the PDSA model to train other clinic faculty physicians. The chair of the department conducted several telehealth clinic visits for sleep disorder patients a week before the launch date and shared his experiences with all the clinic faculty physicians. The process of physician training was focused on 2 components: (1) learning the telehealth clinic process and obtaining patient's consent after explanation and (2) learning the methods of clinical evaluation in telehealth visits, especially ways of performing physical examination.

## 5. Learning the Telehealth Clinic Process

All clinic faculty physicians completed an online training provided by the University of Missouri Health Care reviewing the best practice guidelines to conduct a telehealth appointment and to enhance patient experience. Providers were educated on best practices for sound and light optimization to ensure a successful telehealth encounter. After completion of the online training part, the associate medical director conducted a 1 : 1 training session with a group of physicians to get them comfortable with video visit experience. To ensure a smooth process, for the first couple of weeks, only follow-up visits in selected neurological subspecialty areas (headache, sleep, stroke, and epilepsy) were scheduled. Follow-up patients did not require extensive neurological examination as compared to new patients, and some encounters were focused on symptom management which was relatively easy to do through telehealth. Providers were required to establish a University of Missouri system protected, HIPAA compliant Zoom account to conduct telehealth visits. At commencement of each telehealth visit, providers were required to discuss the risk and benefits of the virtual encounter emphasizing the importance of privacy and patient safety. They were required to document the patient's consent for virtual visit prior to starting the encounter. Physicians were trained to use specialized telehealth/Zoom note type to document patient encounters based on recommendations from the American Academy of Neurology (AAN) [16].

As per CMS guidelines for physicians at teaching hospitals (PATH) working with residents, the teaching physicians were required to be present virtually during the key and critical portions of the service and addend the notes demonstrating the teaching physician's presence, including the services provided via telehealth [17]. Prior to starting telehealth appointments, trainees including residents and fellows were required to complete online training modules for physicians designed by the University of Missouri Health Care. Following completion of online modules by all trainees, the program director conducted 1 : 1 meeting with the chief residents and performed simulated virtual patient visit encounters. The chief residents then performed the same with different groups of residents to get them accustomed to virtual clinic visit experience. The chief residents took a lead and conducted their first telehealth clinic visit before other residents. All other residents started conducting telehealth appointments in the first week of May, 2020.

### 5.1. Learning the Methods of Clinical Evaluation

We utilized guidelines proposed by the American Academy of Neurology (AAN) to help our physicians perform quick but thorough neurological examination during telehealth encounters [18].  Table 1 describes the details of neurological examination conducted during telehealth appointments.

## 6. Ensuring Adequate Hardware and Software Support

As per CMS guidelines, to conduct a telehealth visit, providers were required to have an interactive audio and video telecommunication system that would permit real-time communication between the provider at the distant site and the beneficiary at the originating site [17]. Though CMS allowed the use of nonpublic facing platforms, the University of Missouri Health Care system operationalized a HIPAA compliant Zoom for health care platform to conduct telehealth visits. This platform allowed video and/or audio connections without exposing the provider's personal phone number, e-mail, or other information. Our neurology clinic has 20 patient encounter rooms with desktop computers but not all of them had built-in video cameras and interactive audio setup. The providers were advised to use their personal phones, laptop, or other electronic devices to login their UM system protected HIPAA compliant Zoom for health care platform and conduct a telehealth visit. To ensure adequate software support and provide assistance with video operational issues, a dedicated Zoom helpline desk was established by the University of Missouri Health Care.

We initially anticipated that providers might need specialized headsets for optimal audio input but with the first few telehealth visit experiences, personal electronic equipment was found to be equally effective. Providers were instructed to use the patient encounter room in the neurology clinic or their office rooms and keep the door locked during the virtual encounter to eliminate background noise and maintain patient privacy and HIPAA compliance.

## 7. Implementation and Outcomes

Following completion of training of all PSRs, nurses, and providers and after ensuring adequate hardware and software support, the department decided a soft launch date of April 1, 2020. The initial plan was to roll out follow-up video visits across providers within different subspecialties which are relatively slow.

**Figure 4:**
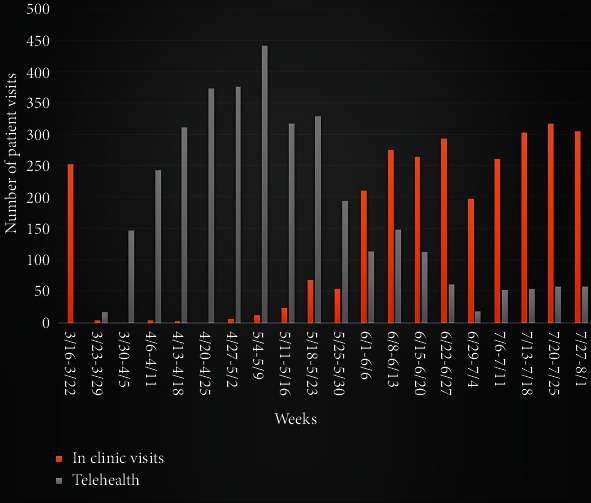
Total number of visits conducted at the neurology clinic from 3/16/20 to 8/1/2020.

The state of Missouri activated stay at home order on 3/24. The chair and associate medical director conducted pilot telehealth clinic visits from 3/17 to 3/30 to optimize workflow and identify changes needed to improve. During 1st week of implementation (3/30-4/5), 147 telehealth visits were conducted. As providers got accustomed to using the virtual platform, over the next few weeks, the number of telehealth visits increased gradually (Figure 4). With 18 active clinic providers conducting telehealth visits, for the month of April, 2020, the Department of Neurology completed 1451 telehealth visits which was the 2nd highest number of telehealth visits conducted by any department in the University of Missouri-Columbia Health Care system, next only to the Department of Family and Community Medicine which has more than triple the number of providers than the neurology department.

During the statewide stay at home order, the University of Missouri Health Care system worked extensively to ramp up the personal protective equipment (PPE) supplies and testing abilities to ensure safe return of in-person clinic visits. The university worked with voluntary organizations to develop supplies of hand-stitched masks that could be used during in-person clinic visits. On May 3rd, 2020, the state of Missouri lifted the stay at home orders. By that time, the University of Missouri Health Care system had stockpiled enough reserves of hand-stitched masks and other PPEs to restart in-person clinic visits safely. To ensure patient and staff safety, screening checkpoints were established at clinic entrances where everyone entering the facility was required to undergo temperature checks and was checked for symptoms related to COVID-19 infection (cough, shortness of breath, fever, chills, muscle aches, vomiting, diarrhea, and new loss of sense of smell or taste), as defined by Centers of Disease Control (CDC) [19]. A strict no-visitor policy was implemented throughout the entire University of Missouri Health Care system.

Starting week of 5/4-5/9, the neurology clinic started scheduling in-person clinic visits in addition to telehealth visits. For the month of May, 2020, a total of 1440 patients were seen in neurology clinic. 89% of all patients (*n* = 1283) were seen via telehealth. The department gradually started scheduling more in-person visits to meet patient needs and optimize the workflow. For the month of June, 2020, a total of 1480 patients were seen in neurology clinic. 29% of all patients (*n* = 439) were seen via telehealth, whereas 71% (*n* = 1045) were seen physically in the clinic. Figure 4 depicts the number of patient visits conducted at the neurology clinic from March 16, 2020 to August 1.

## 8. Discussion

The Neurology and Sleep Disorders Clinic at the University of Missouri-Columbia successfully transitioned all in-person clinic visits to telehealth virtual visits in a span of 2 weeks with collaborative effort of PSRs, nursing staff, clinic physician providers, and the leadership. Within a month of soft launch, 18 clinic providers conducted 1451 telehealth visits which was the 2nd highest number of telehealth visits conducted by any department at the University of Missouri-Columbia Health Care system.

Looking back, the key factors for our success were dedicated clinic staff, physician engagement, and robust support of the leadership. The chair of the department led from the front and conducted several pilot telehealth clinic visits before the official launch date and addressed several technical, logistical, workflow, and equipment-related issues. After initial deployment of the process, to ensure smooth implementation of workflow, the associate medical director and chair held several meetings with the clinic providers, residents, and fellows and frequently visited schedulers' workstations in person to address their concerns. Some of the factors that contributed to our success were as follows: (1) scheduling only follow-up visits during the first few weeks to get providers accustomed to the process, (2) having PSRs and nursing staff call the patient in advance to get them acquainted to the telehealth experience, and (3) implementation of teach back technique by every staff member to consolidate the learned objectives.

Lack of connectivity, poor video/audio quality, and unavailability of smart devices among rural populations were the important shortcomings identified during our telehealth experience. We were able to overcome these shortcomings to a certain extent by utilizing out excellent Zoom technology support staff and in certain cases by conducting a phone call visit. Nonetheless, such visits compromised certain components of neurological examination. Digital divide continues to remain a hot topic in the state of Missouri. In 2017, Senator Blunt reported, “61 percent of rural Missourians don't have access to high speed internet” [20]. Lower broadband width and slower internet speed leaves rural Missourians at a health care and economic disadvantage [20]. There is a strong need for expansion of high-speed internet access across rural Missouri.

Many patients with neurological diseases suffer physical or cognitive difficulties, rendering them dependent on caregivers to get to and from clinics [3]. In addition, patients with cognitive difficulties may experience difficulties in operating smart devices. Certain neurological conditions may require in detail examination which may not be possible on a virtual platform. Neurological procedures including sleep studies, Botox injections, electrodiagnostic studies, and nerve blocks all require in-person visits. Another major long-term limiting factor that might discourage long-term use of telemedicine is termination of compensation for telehealth visits and issue of lack of reimbursement of facility fees.

On the other hand, studies have shown that telehealth visits can potentially save time and travel costs. In a veteran's study, telemedicine visits resulted in an average travel savings of 145 miles and 142 min per visit [21]. This led to an average travel payment savings of $18,555 per year [21]. Telehealth visits can potentially expedite posthospital discharge follow-up wait time. Telehealth visits not only prevent vulnerable patients from unnecessary exposure at health care facilities but also potentially save patients and/or their caregivers' travel time and associated out-of-pocket and health care costs [3, 4, 7, 22, 23].

Currently, telehealth visits account for approximately 20% of all our clinic visits. We are constantly reviewing change in new COVID-19 cases and adjusting our clinic scheduling accordingly. Going forward, there is a huge need to bridge the digital divide and introduce long-term Medicare reforms in telehealth to ensure high quality and easily accessible health care to our patients.

## 9. Conclusion

Telehealth, including teleneurology, was already being tried for selective areas such as telestroke to provide care to communities with limited local provider resources. However, COVID-19 enhanced this process, evolving its application to neurological areas beyond stroke. Through the collaborative effort of our dedicated clinic staff, physician engagement, and robust support of the leadership, we successfully transitioned to telehealth visits across multiple neurological subspecialties within a span of 4 weeks. Our experience highlighted poor internet connectivity as one major shortcoming leaving rural Missourians at a distinct health care disadvantage. We believe telehealth is a future mode of medical/neurological care to serve our communities, especially those with lack of expertise and/or medical providers. This is one effective way to deliver the expertise of neurological care, which is predominantly provided in a health care facility, directly on site to the patients in place of patients coming to a facility.

## Figures and Tables

**Table 1:** Detailed neurological examination performed during telehealth appointments.

Examination	Procedure	Family member needed
Vitals	Patients check their own heart rate, temperature, and blood pressure.	
Mental status	Minimental state examination Montreal cognitive assessment blind version	
Speech and language	Asked to repeat words and sentences after the examiner (Pa, Ta, Ka, today is a bright sunny day, British constitution) Asked to follow multiple step commands Examiner would hold a language card and ask the patient to read a sentence. Write a sentence from a language card and show it to the examiner on screen. Name an object selected by the examiner from the language card.	
Cranial nerves	CN I: asked to smell coffee beans, one nostril at time CN II: patients were asked to hold a flashlight/penlight behind the computer screen and shine light directly in center of eyes while looking directly at the camera. Pupillary size and reactivity were documented using Zoom in option on Zoom. CN III, IV, and VI: extraocular movements were assessed by asking patients to look in 6 cardinal directions of gaze (right/up, right, right/down, left/up, left, and left/down). Patient fixates on camera and rotates head from side to side for fixation. Smooth pursuit: look left and then look right. Saccades: patients would keep their head still and look back and forth between an object on their left and another object on their right and then the ceiling and the floor. Convergence: hold a pen in front of their face and watch it as they slowly move it towards their nose. Examiner would hold a phone with the optokinetic nystagmus application towards the camera and check for nystagmus with strips moving in different directions (right, left, up, and down). CN V: motor: patients were asked to open mouth, and examiner would look for jaw deviation. Patients were asked to clench their teeth tight, and the examiner would look for masseter and temporalis atrophy. CN VII: patients were asked to close their eyes tightly, wrinkle forehead, contract platysma, smile, and puff cheeks. CN VIII: patients were asked to rub fingers near each ear and check and compare if they are able to hear noise well and equal on both sides. CN IX and X: patients were asked to open their mouth in front of the camera, and the examiner would check for tongue atrophy or fasciculation. Patient was asked to stick out his tongue and say “ahhh,” and the examiner would check for palate movement. CN XI: patients were asked to place their hand on their cheek and then try to turn their head to the same side, and the examiner would look for sternocleidomastoid contraction. Patients were asked to shrug their shoulders. Examiner would look for sternocleidomastoid contraction. CN XII: patients were asked to open their mouth in front of the camera, stick out tongue, and move it side to side.	CN VIII: family members were asked to rub fingers near each ear and check and compare if the patient is able to hear noise well and equal on both sides.
Motor examination	Bulk: observed while examining each extremity Range of motion: patient was asked to perform active range of motion movements at each joint. Strength: performed in detail with family members nearby, and if it is not available, the examiner would observe muscle activation against gravity with range of motion movements. Involuntary movements: examiner looks closely for bradykinesia, resting tremor, postural tremor, fasciculation, and other involuntary movements. Rapid alternating movements: finger tapping, finger rolling, arm rolling, and foot tapping	Family members were asked to provide resistance and observe muscle activation while the patient performed range of motion movements at each joint.
Sensory	Performed with help of family members, if possible	
Cerebellar	Patients were asked to extend their arm all the way out, then touch the index finger of the extended hand with the index finger of the opposite hand and then to their nose, and then repeat the same on the opposite side. The patient was asked to perform heel to shin on each side.	
Gait	Performed only if the patient was comfortable and/or a family member was available for support, if needed Patient was asked to stand from a sitting position without using support. After adjusting the camera for optimum view for the examiner, the patient was asked to perform tandem walks, walk on toes and heels. With a family member nearby, the patient was asked to stand with feet together with eyes closed to look for Romberg's sign.	Family members were provided with support during gait examination ensuring patient's safety.
Reflexes	Difficult to assess	

## Data Availability

The data used to support the findings of this study are included within the article.

## Abbreviations

FIN: Facility Identification Number
AAN: American Academy of Neurology
PSRs: Patient service representatives
CMS: Center of Medicare and Medicaid Services
PDSA: Plan Do Study Act
PATH: Physicians at teaching hospitals.
